# Ultrasonography-guided removal of plant-based foreign bodies from the lacrimal sac in four dogs

**DOI:** 10.1186/s12917-019-1817-9

**Published:** 2019-03-05

**Authors:** Giovanni Barsotti, Tommaso Mannucci, Simonetta Citi

**Affiliations:** Department of Veterinary Science, University of Pisa, via livornese snc, San Piero a Grado, 56122 Pisa, Italy

**Keywords:** Dacryocystitis, Dog, Ocular discharge, Nasolacrimal system, Plant-based foreign body, Ultrasound

## Abstract

**Background:**

Dacryocystitis is an inflammation of the lacrimal drainage system. One of the most common causes of dacryocystitis in dogs is due to plant-based foreign bodies typically located in the lacrimal sac. The aim of this case series was to describe an ultrasonography-guided technique for dogs with plant-based foreign bodies in the lacrimal sac, as both a diagnostic and therapeutic tool.

**Case presentation:**

Four dogs with clinically suspected plant-based foreign body in the lacrimal sac (with a total of five eyes affected) were evaluated by ultrasound with a multifrequency (8–14 MHz) linear probe. Under general anesthesia, the foreign body was removed using Hartmann alligator forceps inserted thorough the upper puncta. Ultrasound was used to guide the forceps in grasping the foreign body. Ultrasound was positive in four out of five lacrimal sac diseases. All identified foreign bodies were successfully removed by the ultrasonography-guided technique.

**Conclusions:**

The results show that ultrasound is a fast, non-invasive, and inexpensive method for the assessment of dacryocystitis due to foreign bodies in dogs. Ultrasound is also useful not only for identification, but also in the non-invasive removal of the foreign body from the lacrimal sac. To the best of authors’ knowledge, this is the first study to describe the sonographic approach to the palpebral medial cantus as an initial diagnostic step in canine dacriocystitis.

**Electronic supplementary material:**

The online version of this article (10.1186/s12917-019-1817-9) contains supplementary material, which is available to authorized users.

## Background

Dacryocystitis is an inflammation of the lacrimal drainage system, specifically of the lacrimal sac [1]. The disease is usually unilateral, and in the canine species, it can affect animals at any age. Is characterized by a mucoid to mucopurulent discharge, which is particularly evident at the level of the medial palpebral cantus. Moderate pressure on the skin over the lacrimal sac can result in reflux of mucopurulent material through the lower punctum. One of the most common causes of dacryocystitis in dogs is due to plant-based foreign bodies typically located in the lacrimal sac [1]. Foreign bodies have been rarely reported in the nasolacrimal duct [2, 3]. The diagnostic challenge in the management of dacryocystitis is to understand whether or not a foreign body is really present. Contrast dacryocystorhinography as well as contrast computed tomography could be useful in demonstrating a complete or partial nasolacrimal obstruction. However, these procedures do not always lead to an etiological diagnosis [4–6]. Retrograde or normograde lavages of the lacrimal drainage system can also be used for both diagnosis and therapy [7].

The purpose of this study was to present four cases of dacryocystitis due to a foreign body, which presented to our department within a 1-year time period, and to report on an alternative approach for the diagnosis and management of the disease. In particular, ultrasonographic identification and non-surgical ultrasonography-guided removal of the foreign bodies from the lacrimal sac are outlined below.

## Case presentation

The four dogs described below underwent a complete ophthalmic examination, which included a Schirmer tear test (Dina strip Schirmer-Plus, GECIS sarl, France), basic neurophthalmic assessment, slit-lamp biomicroscopy (SL-17, Kowa Company, Japan), rebound tonometry (Tonovet®, Icare, Finland), and fundus ophthalmoscopy (Omega 180, Heine, Germany). Fluorescein staining was also performed.

### Case 1

A 1-year old, female, mixed-breed dog was referred with unilateral, mucopurulent discharge from the left eye of 14 days’ duration. The ocular problem was acute in onset and developed after a walk in a meadow. The referring veterinarian had prescribed 0.3% tobramycin eye drops some days previously, but no ocular improvement had been apparent during this therapy. At ophthalmic examination, the dog showed an abundant mucopurulent to haemorrhagic discharge and a moderate conjunctival hyperemia in the left eye. No other ocular abnormalities were observed in this eye. The right eye did not show any abnormalities. The presumptive diagnosis was unilateral dacryocystitis due to a foreign body.

### Case 2

An 8-year-old, male, English setter was referred for bilateral conjunctivitis treatment. The ocular problem had been present for at least 12 months, and had started at the end of the hunting period. Unspecified topical antibacterial therapy had previously been performed. The owner had seen no improvement during this therapy. An abundant mucopurulent discharge associated with a severe conjunctival inflammation and a mild ocular mucous discharge with conjunctival hyperemia were observed in the left and right eyes, respectively. Nucleosclerosis was present in both eyes and ophthalmoscopic signs of a previous focal chorioretinitis were detected in the left eye. A presumptive diagnosis of bilateral dacryocystitis of unknown origin was made.

### Case 3

A 11-year-old, neutered male, Shih-tzu was presented with bilateral severe chronic ocular problems. The left eye showed buphthalmos, intraocular pressure elevation (35 mmHg), and chronic exposure keratitis with neovascularization and pigmentation. The problem started around 4 years prior to the ophthalmic examination, and no drug protocol had been previously performed in this eye. In the right eye, a moderate mucopurulent discharge, conjunctival hyperemia and superficial keratitis were present. Two fistulas were also detected, one in the margin of the right lower eyelid close to the medial cantus, the second on the skin at the level of the frontal region, between the two eyes. The problem of the right eye started with an ocular discharge 18 months prior to the ophthalmic examination, and the palpebral and skin fistulas had been observed for 6 and 4 months, respectively. The owner was unaware about the possible cause, and no drug protocol had been previously performed also in this eye.

Chronic glaucoma of the left eye, and suspicious complicated dacryocystitis of the right eye were diagnosed. On the basis of Schirmer tear test (STT)-1 readings, a diagnosis of moderate keratoconjunctivitis sicca was also formulated in the right eye.

### Case 4

A 4-year-old, female, Labrador retriever had an abundant mucopurulent and hemorrhagic discharge from the left eye of 7 months’ duration. The ocular problem was acute in onset and developed after a walk in the park. A conjunctival hyperemia and mild chemosis were also present. No other ocular abnormalities were observed in this eye. In the right eye ocular abnormalities were not found. The presumptive diagnosis was unilateral dacryocystitis of unknown origin.

A preliminary ultrasound of the palpebral medial cantus was performed in the eyes with the presumptive diagnosis of dacryocystitis to examine the superficial portion of the nasolacrimal system, before its entrance into the lacrimal bone. No attempts to flush the nasolacrimal system were performed before the ultrasonographic evaluation. An ultrasonographic device (Aplio 400, Toshiba, Japan) with multifrequency linear probe (Mhz 8–14) was used. The dogs were only manually restrained, and placed in sternal recumbence. The eye was maintained closed, and ultrasound gel was placed between skin and transducer surface. The area was examined by B-mode scanning in the sagittal and cross-sectional planes. In four out of the five examined eyes, a foreign body in the lacrimal sac was identified by ultrasound. In fact, in the case where the dacryocystitis was bilateral (case 2), the foreign body was identified only in the left lacrimal sac. The foreign bodies always appeared as linear spear-shaped hyperechoic structures with dimensions variable from 0.6 cm to 1.8 cm. In all cases a hypoechoic halo attributable to inflammatory fluid was present. In case 1 an edge shadowing originating in the surface of the hypo/anechoic tissue around the foreign body was identified (see Additional file 1). In case three a draining tract between the lacrimal sac and the frontal region was also observed.

After identification of the foreign bodies, the dogs were anesthetized and, under ultrasonographic guidance, a Hartmann alligator forceps was inserted through the upper *puncta*, and directed toward the foreign body. The forceps was opened and the foreign bodies grasped and pulled out (see Additional file 2). At the end of each procedure, a normograde lavage of the nasolacrimal system with 0.9% saline solution was performed. Topical tobramycin (Stilbiotic 0.3% eye drops, Ceva, Italy) q6h for 7 days, amoxycillin /clavulanic acid (Synulox, Zoetis Italia S.r.l., Italy) 12.5 mg/kg q12h PO for 7 days, and carprofen (Rimadyl, Pfizer Italia S.r.l., Italy) 2 mg/kg q24h PO for 7 days were prescribed. At the 21-day follow-up, ocular signs of dacryocystitis had disappeared in cases 1, 3, and 4. No information was available for case 2 because the dog was lost to follow-up.

**Fig. 1:**
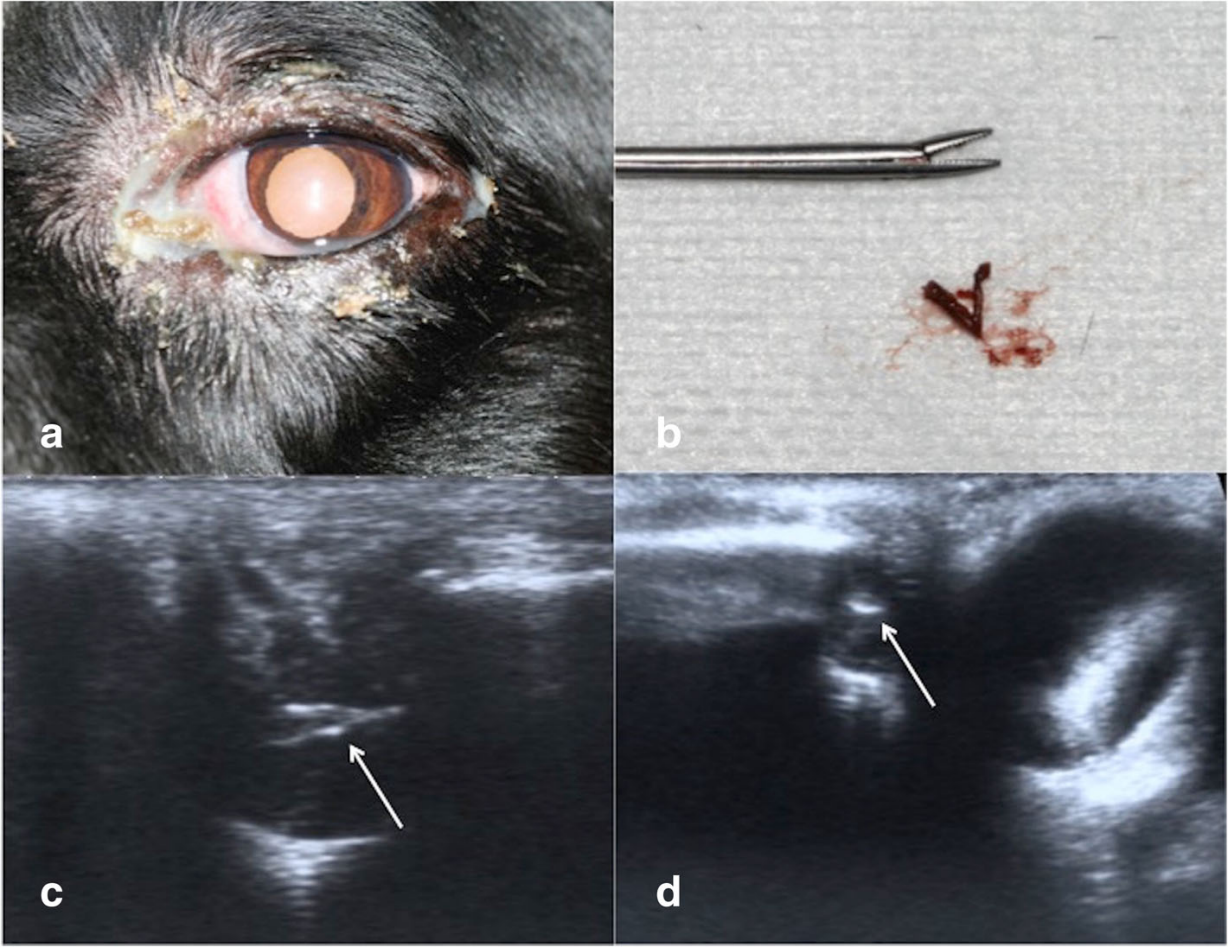
**a** One of the treated dacryocystitis in the left eye of an 8-year-old, male, English setter at presentation (case2). Mucopurulent discharge and conjunctival hyperemia of the third eyelid are evident. **b** The plant-based foreign body removed from the nasolacrimal sac of the dog in Fig. 1a. **c** Ultrasonographic appearance of the plant-based foreign body of Fig. 1b in the sagittal scan (white arrow). The foreign body appears as a linear spear-shaped hyperechoic structure in the lacrimal sac. **d** Ultrasonographic appearance of the plant-based foreign body of Fig. 1b in cross-sectional scan (white arrow). The foreign body appears as a hyperechoic rounded cross-sectional structure, medial to the left eye

**Fig. 2:**
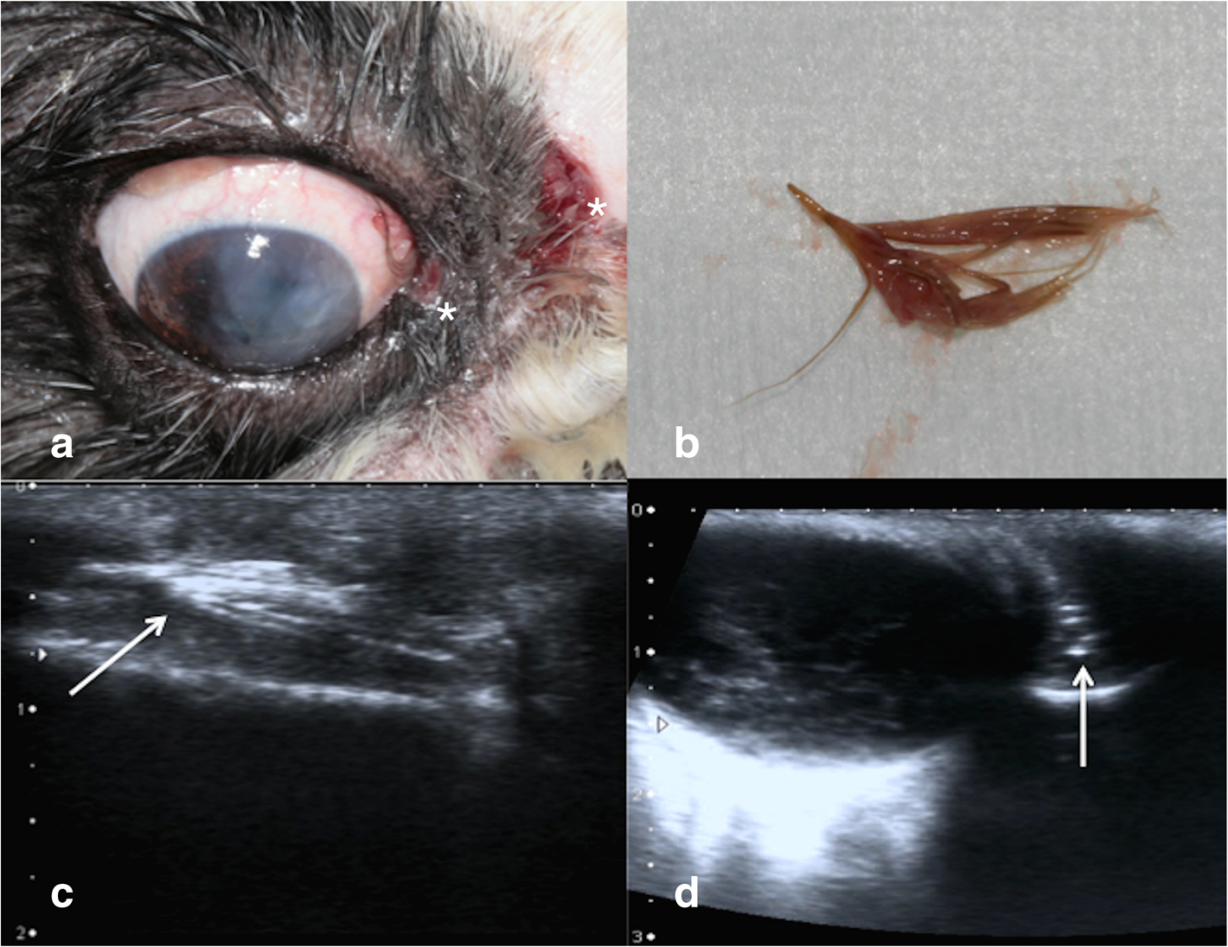
**a** One of the treated dacryocystitis in the right eye of a 11-year-old, male, Shih-tzu at presentation (case 3). Two fistulas are present, one in the margin of the lower eyelid close to the medial cantus, and the second on the skin at the level of the frontal region, as indicated by *. A mucopurulent discharge is not evident because it was removed before taking the picture, in order to show the two fistulas. Note the keratitis due to keratoconjunctivitis sicca. **b** The plant-based foreign body removed from the nasolacrimal sac of the dog of Fig. 2a. **c** Ultrasonographic appearance of the plant-based foreign body of Fig. 2b in the sagittal scan (white arrow). It appears as spindle-shaped hyperechoic foreign body. **d** Ultrasonographic appearance of the plant-based foreign body of Fig. 2b in the cross-sectional scan (white arrow) in the lacrimal sac. The foreign body appears as a hyperechoic rounded cross-sectional structure, medial to the eye

The cases of the English setter (*case 2*) and the Shih-tzu (*case 3*) are reported in Figs. 1 and 2, respectively.

## Discussion and conclusions

This case series concerned the ultrasonographic identification of four foreign bodies in the lacrimal sacs of dogs affected by dacriocystitis. Clinically, our cases did not exhibit peculiarities, with the exception of the dog of case three. This dog showed two fistulas, one in the margin of the right lower eyelid close to the medial cantus, the second on the skin at the level of the frontal region. The draining tract between the lacrimal sac and the fistula in the frontal region was also identified by ultrasonography. The 18 months’ duration of the dacriocystitis without any treatment may justify the presence of fistulas.

To the best of our knowledge, this is the first study to describe the sonographic approach to the palpebral medial cantus as an initial diagnostic step in canine dacriocystitis. Contrast dacryocystorhinography is commonly used to identify and locate nasolacrimal system obstruction in eyes presenting with ocular discharge [4, 5, 8]. Computed tomographic-dacryocystorhinography can be an alternative, advanced technique that avoids the superimposition of many structures present in conventional contrast radiography [9]. However, both the previously reported procedures do not always lead to an etiological diagnosis of dacriocystitis [4–6].

In our cases ultrasound helped to identify a linear spear-shaped hyperechoic structure with variable dimensions in the lacrimal sac. The possibility of identifying a foreign body is not the only advantage of the diagnostic sonographic approach to dacriocystitis. In fact, at the same time as the diagnostic procedure, the foreign body identified could also be removed by ultrasonographic guidance. Normally, foreign bodies can be flushed from the nasolacrimal duct system by retrograde or normograde lavages. In the case of failure, a dacryocystotomy followed by cannulation of the duct can be performed [10]. Using the ultrasonographic-guided removal of the foreign bodies described in our cases, no invasive surgical procedures were necessary.

In our experience, ultrasound represents a fast, simple, non-invasive, and inexpensive method for the assessment of dacryocystitis due to foreign bodies in the dog. However, ultrasound is applicable only in diseases of the lacrimal sac and canaliculi, because the lacrimal duct runs intraosseously through the nasolacrimal canal, and is inaccessible to ultrasound signals.

In conclusion, performing an ultrasound of the palpebral medial cantus is useful not only in identification, but also in terms of the non-invasive removal of the foreign bodies from the lacrimal sac. However, when the etiology is unclear or in complicated cases, more complex imaging and surgical methods are necessary. Our results suggest that ultrasound can be routinely employed in the diagnostic management of dacryocistitis in dogs as a first-step approach to the disease.

## Additional files

Additional file 1: Movie of the ultrasonographic identification of a plant-based foreign body in the lacrimal sac in a 1-year old, female, mixed-breed dog (*case 1*). The movie shows the eyeball and then the lacrimal sac containing the plant-based foreign body. Firstly the foreign body appears in cross-sectional scan and finally in the sagittal scan. (MOV 11987 kb)

Additional file 2: Movie of the ultrasonography-guided removal of the plant-based foreign body identified in Additional file 1 (*case 1*). On the left it is possible to observe the Hartmann alligator forceps tips directed toward the foreign body. Under ultrasonographic guidance the tips grasp and pull out the foreign body. (MOV 12528 kb)

## Abbreviations

MHz: egahertz
mmHg: millimeter of mercury
PO: by mouth
q12h: every twelve hours
q24h: every twenty four hours
q6h: every six hours
STT: Schirmer tear test
